# Supplementary figures and images for: Evidence for an increase in cannabis use in Iran – A systematic review and trend analysis
Source: PLoS One. 2021 Aug 30;16(8):e0256563. doi: 10.1371/journal.pone.0256563 (PMC8404985; doi:10.1371/journal.pone.0256563)

###
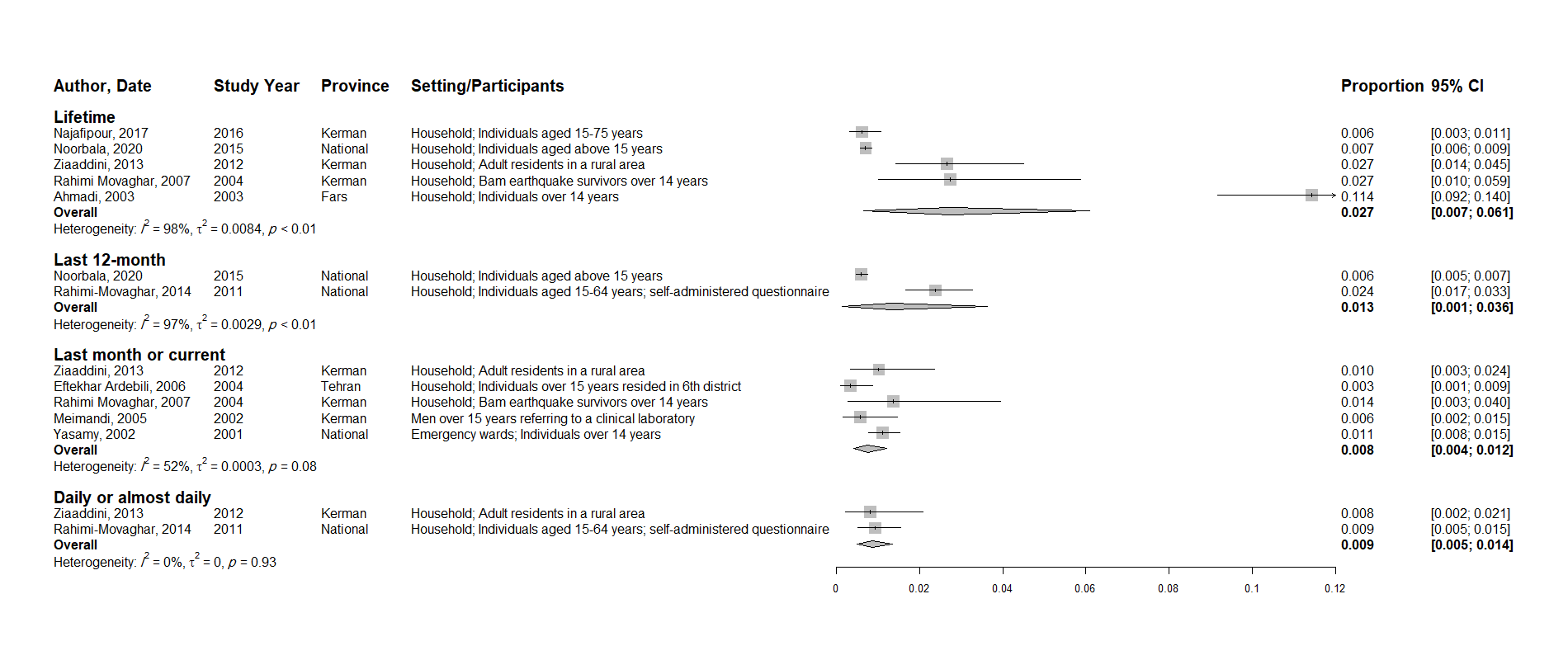
S1 Fig - The pooled prevalence of cannabis use among the male general population

Supplement: S1 Fig — (DOCX) [file pone.0256563.s001.docx]

###
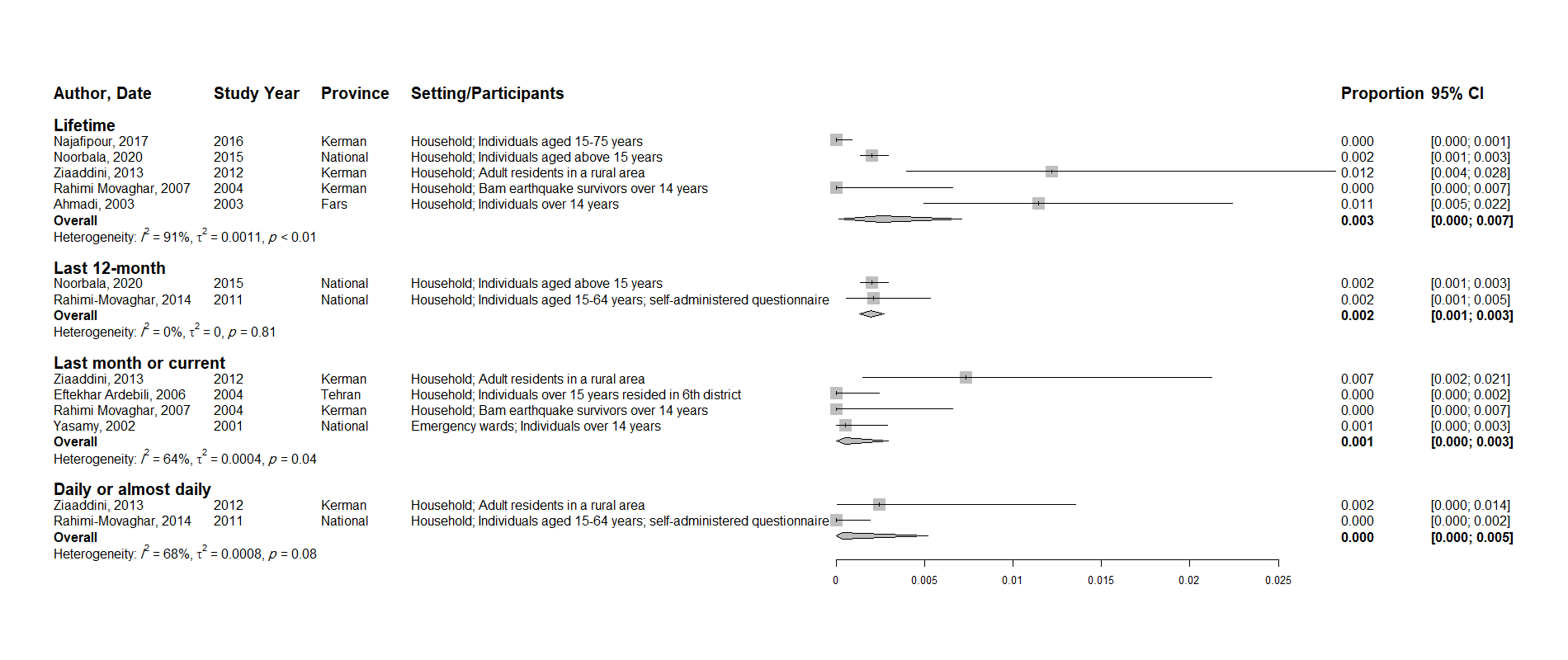
S2 Fig - The pooled prevalence of cannabis use among the female general population

Supplement: S2 Fig — (DOCX) [file pone.0256563.s002.docx]

###
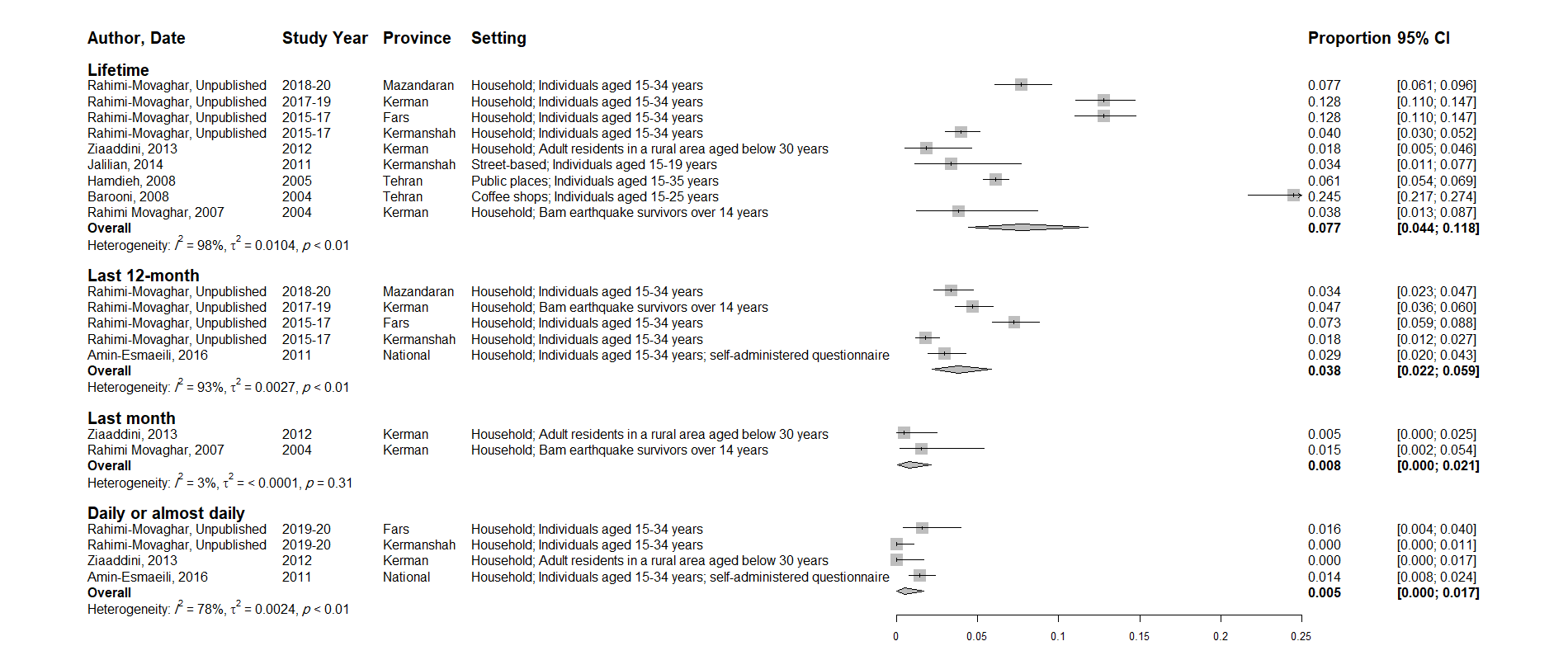
S3 Fig - The pooled prevalence of cannabis use among the male young general population

Supplement: S3 Fig — (DOCX) [file pone.0256563.s003.docx]

### S4 Fig - The pooled prevalence of cannabis use among the female young general population

**
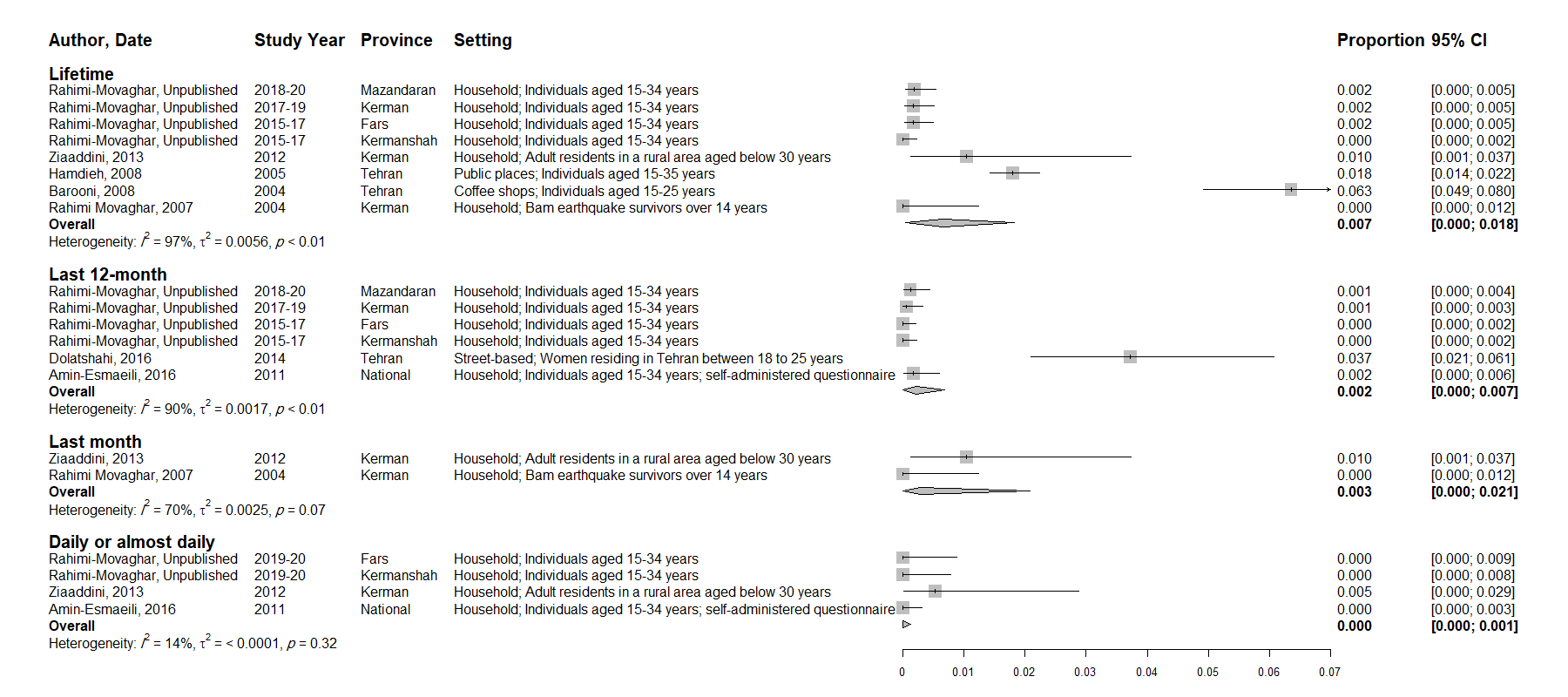
**

Supplement: S4 Fig — (DOCX) [file pone.0256563.s004.docx]

###
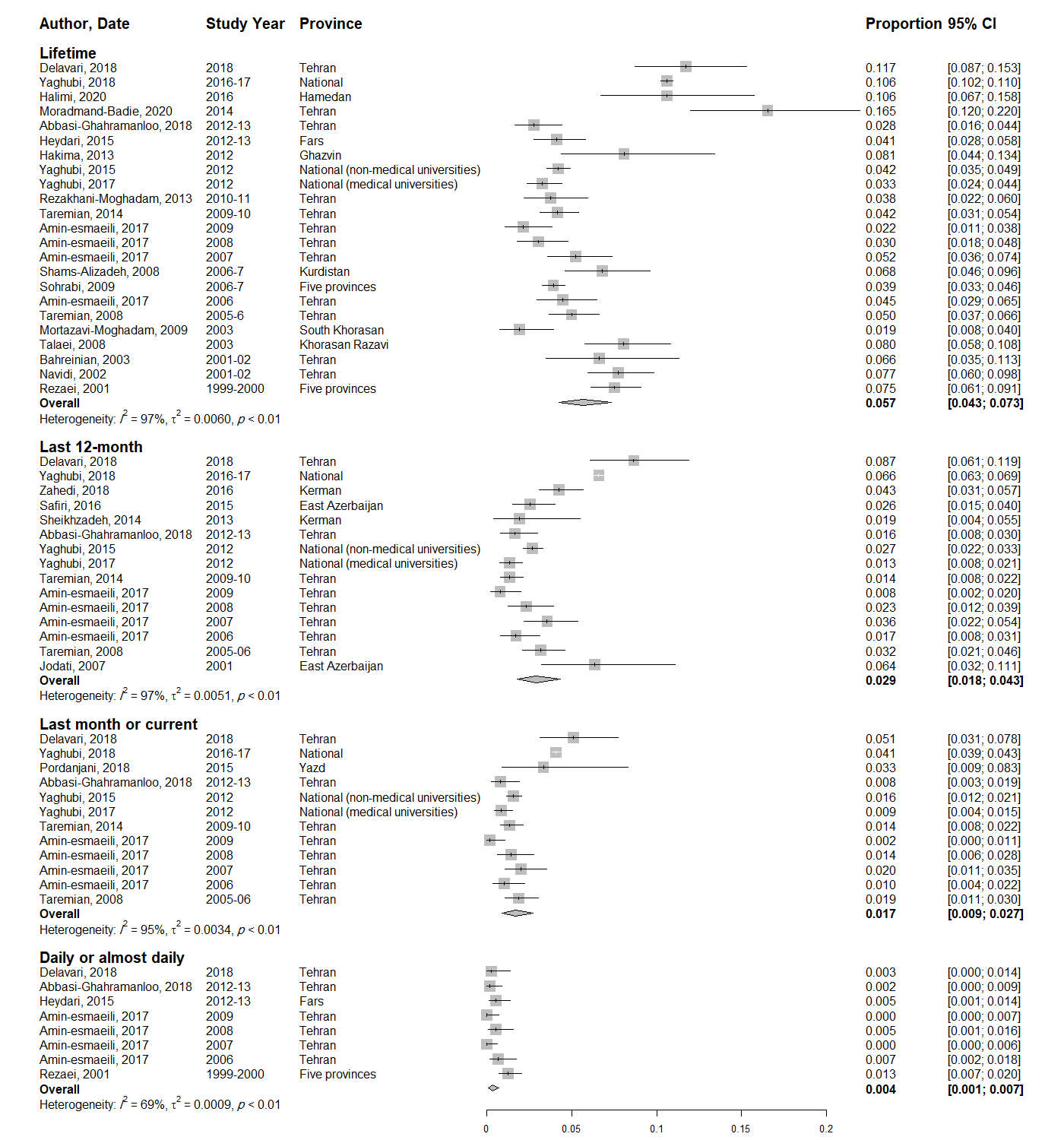
S5 Fig - The pooled prevalence of cannabis use among male university students

Supplement: S5 Fig — (DOCX) [file pone.0256563.s005.docx]

###
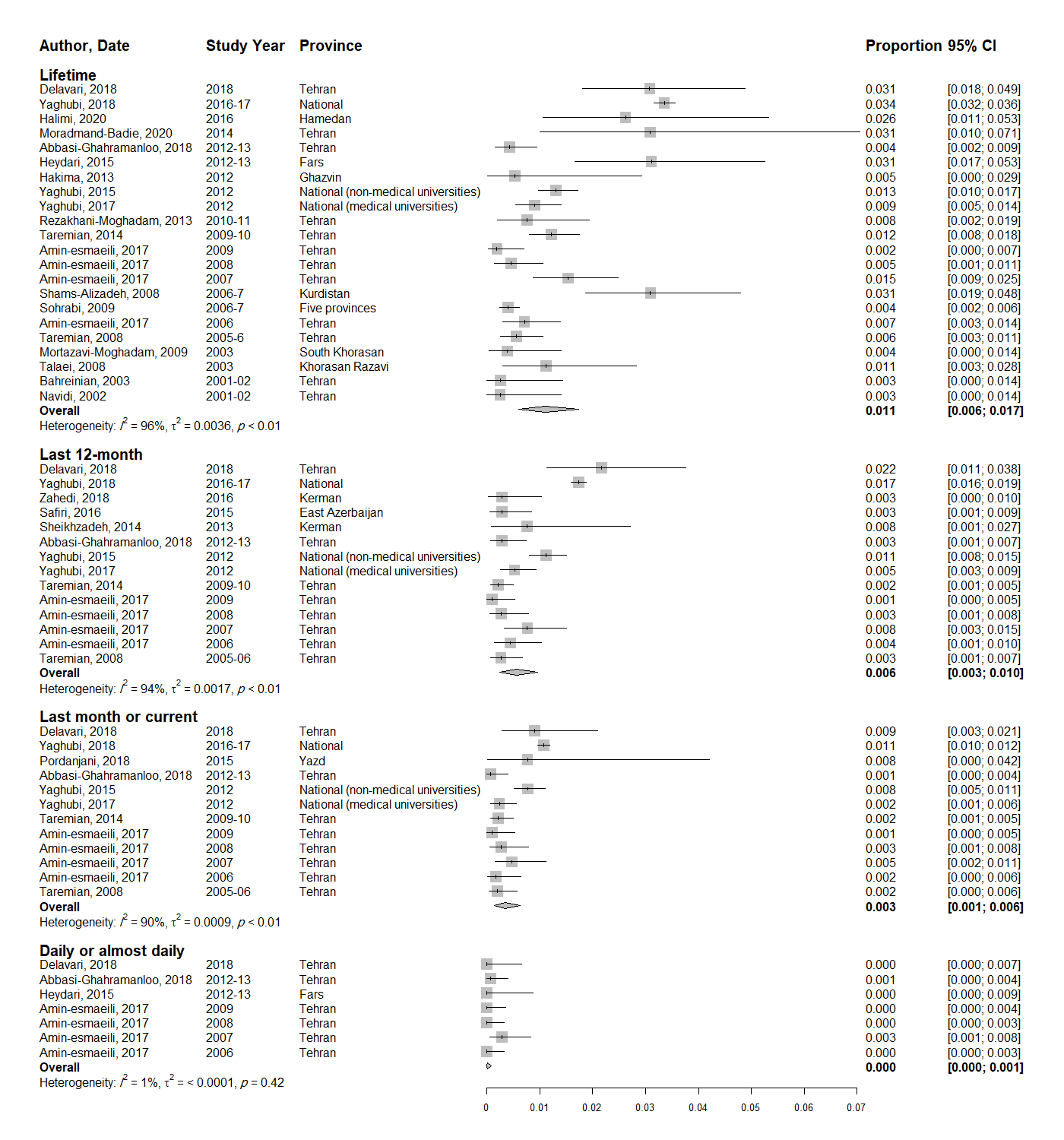
S6 Fig - The pooled prevalence of cannabis use among female university students

Supplement: S6 Fig — (DOCX) [file pone.0256563.s006.docx]

### S7 Fig - The pooled prevalence of cannabis use among male school students

**
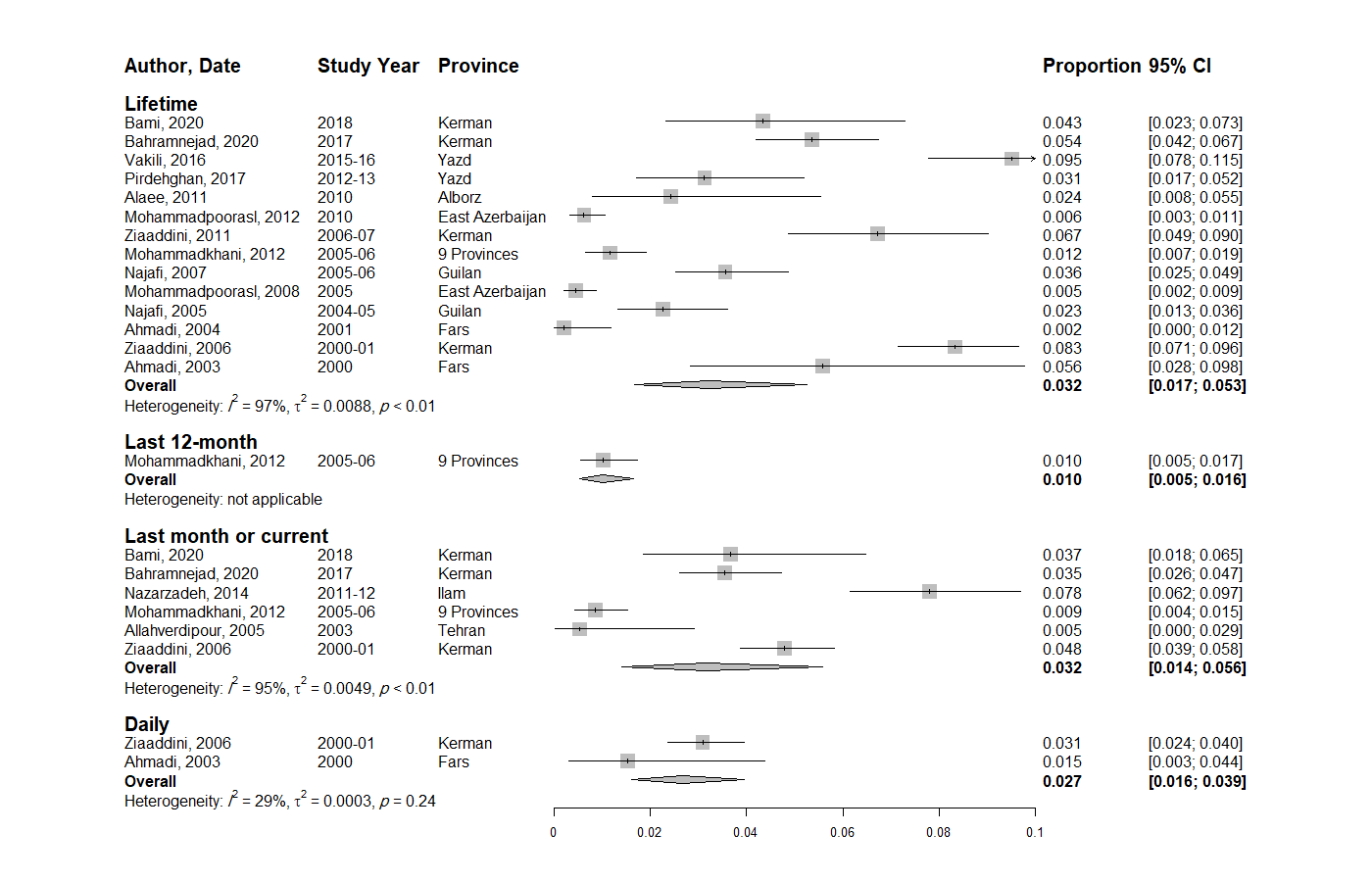
**

Supplement: S7 Fig — (DOCX) [file pone.0256563.s007.docx]

### S8 Fig - The pooled prevalence of cannabis use among female school students

**
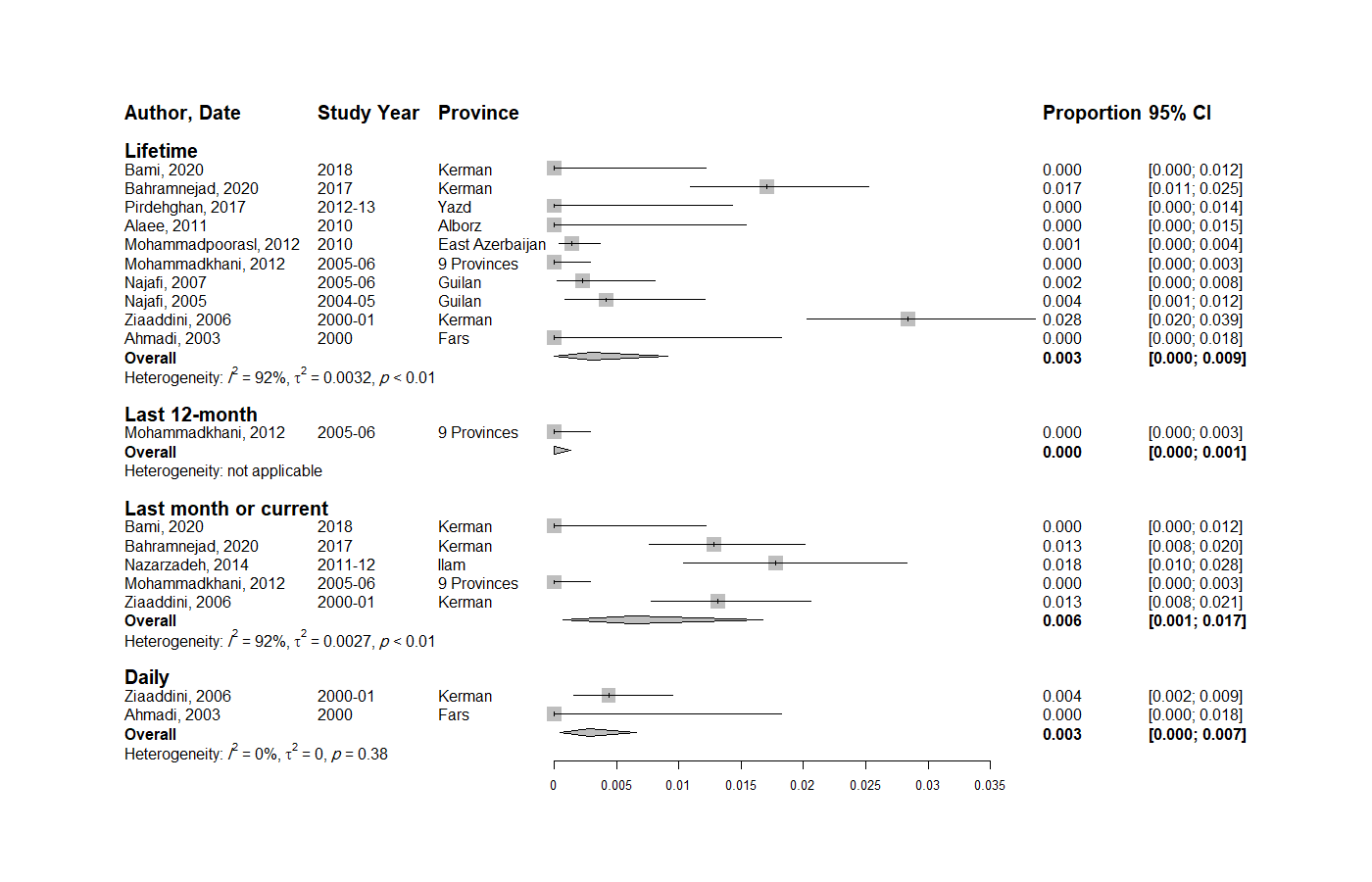
**

Supplement: S8 Fig — (DOCX) [file pone.0256563.s008.docx]

### S9 Fig - The pooled prevalence of cannabis use among people who use drugs

**
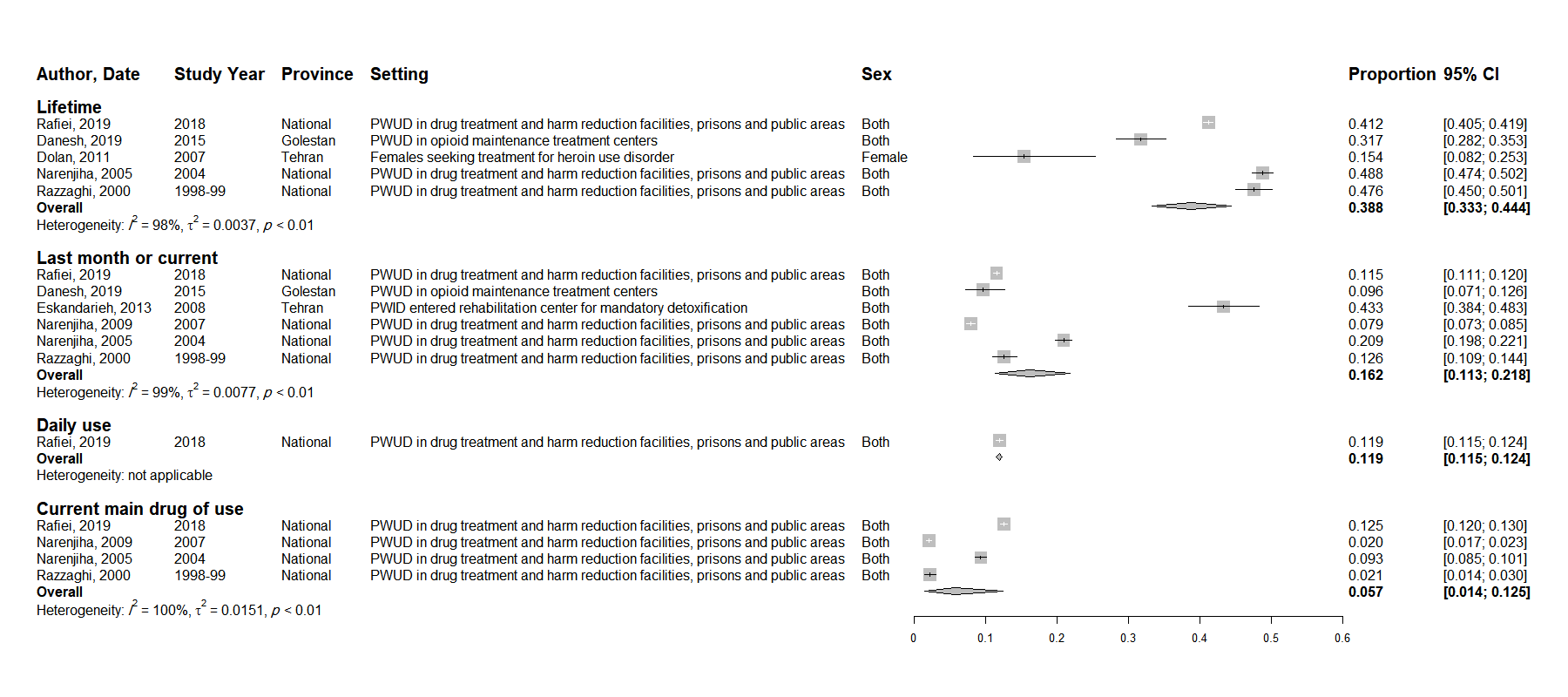
**

Supplement: S9 Fig — (DOCX) [file pone.0256563.s009.docx]

### S10 Fig – The pooled prevalence of lifetime cannabis use among male prisoners

**
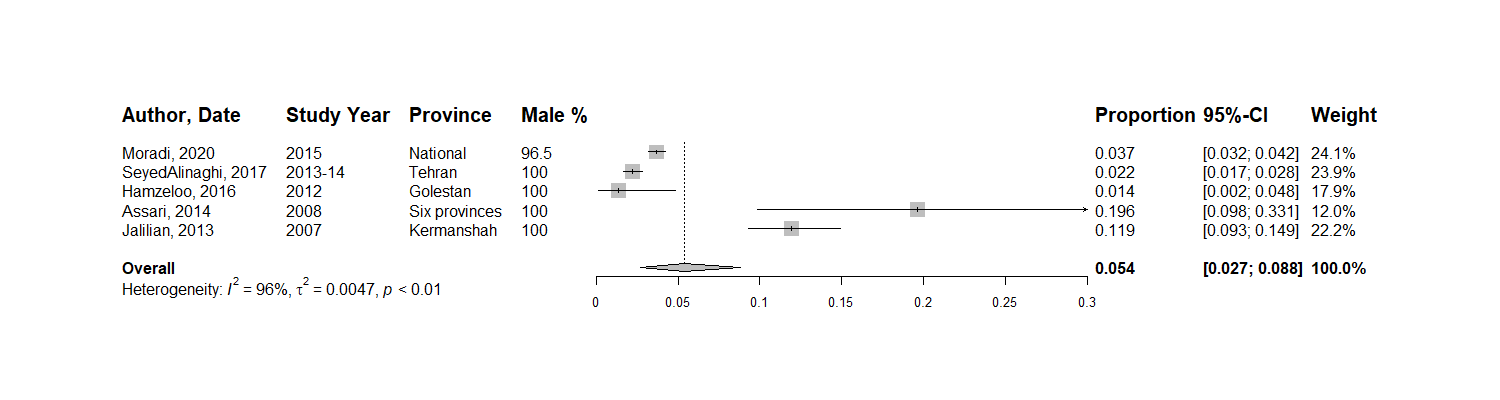
**

Supplement: S10 Fig — (DOCX) [file pone.0256563.s010.docx]
