# Supplementary material for: Evidence for an increase in cannabis use in Iran – A systematic review and trend analysis
Source: PLoS One. 2021 Aug 30;16(8):e0256563. doi: 10.1371/journal.pone.0256563 (PMC8404985; doi:10.1371/journal.pone.0256563)
Supplement: S11 Fig — The pooled prevalence of last 12-month cannabis use among “combined youth groups” in different provinces; a) male subgroup b) female subgroup. The numbers on each province are the pooled estimates and the numbers in the parenthesis are the number of studies. (DOCX) [file pone.0256563.s011.docx]

### S11 Fig– The pooled prevalence of last 12-month cannabis use among “combined youth groups” in different provinces; a) male subgroup b) female subgroup. The numbers on each province are the pooled estimates and the numbers in the parenthesis are the number of studies.


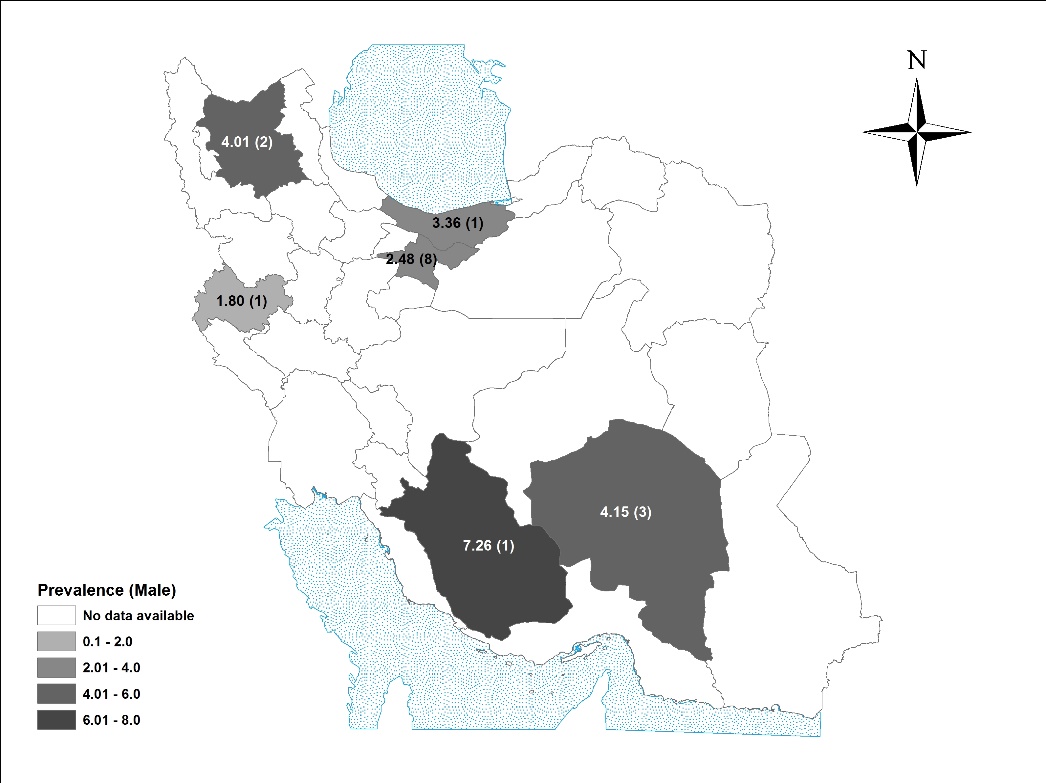


a


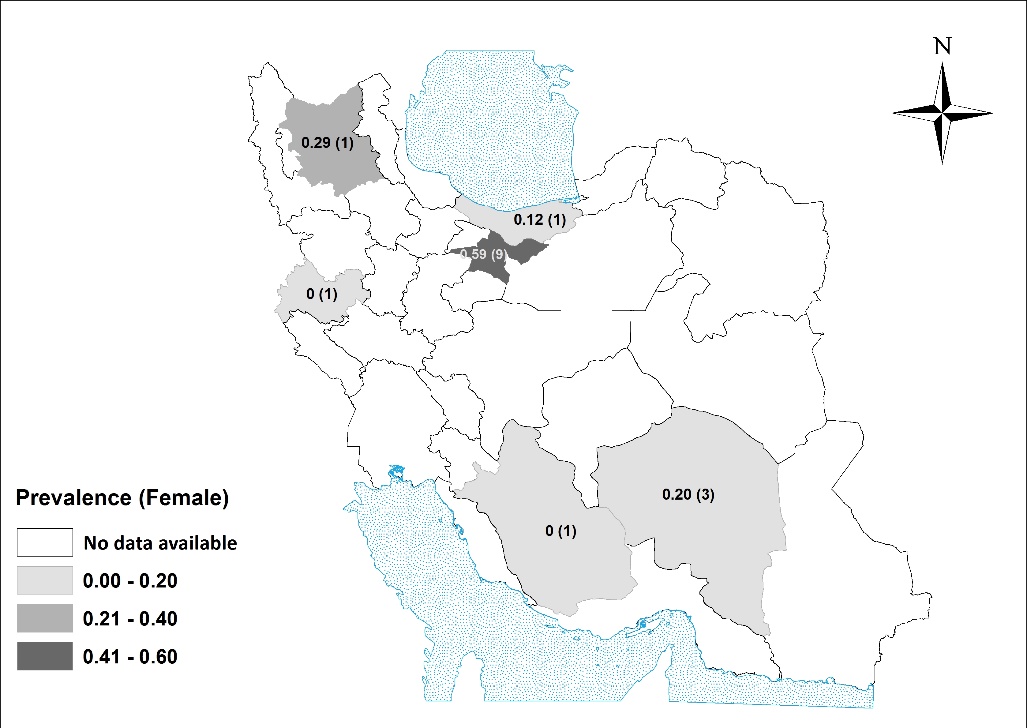


b
