# Supplementary material for: Evidence for an increase in cannabis use in Iran – A systematic review and trend analysis
Source: PLoS One. 2021 Aug 30;16(8):e0256563. doi: 10.1371/journal.pone.0256563 (PMC8404985; doi:10.1371/journal.pone.0256563)
Supplement: S1 Table — (DOCX) [file pone.0256563.s012.docx]

### S1 Tables – Search strategies used in international databases

**The search strategy used in the PubMed database – Search conducted on 16 March 2021**

| **Items found** | **Search query** |  |
| --- | --- | --- |
| **142,273** | (cannabi*[Title/Abstract] OR cannabis[MeSH Terms] OR marijuana[Title/Abstract] OR stimulant drug*[Title/Abstract] OR amphetamin*[Title/Abstract] OR methamphetamine[Title/Abstract] OR methylamphetamine[Title/Abstract] OR ephedrine[Title/Abstract] OR deoxyephedrine[Title/Abstract] OR LSD[Title/Abstract] OR lysergic acid[Title/Abstract] OR cocaine[Title/Abstract] OR ecstasy[Title/Abstract] OR phencyclidine[Title/Abstract] OR PCP[Title/Abstract] OR hallucinogen*[Title/Abstract]) | #1 |
| **190,077** | morphine derivatives[MeSH Terms] OR opium[Title/Abstract] OR morphine[Title/Abstract] OR codeine[Title/Abstract] OR methadone[Title/Abstract] OR narcotic*[Title/Abstract] OR heroin[Title/Abstract] OR opioid*[Title/Abstract] OR opiat*[Title/Abstract] OR tramadol[Title/Abstract] OR hydromorphone[Title/Abstract] OR meperidine[Title/Abstract] OR dihydromorphine[Title/Abstract] OR diphenoxylate[Title/Abstract] OR pentazocine[Title/Abstract] OR buprenorphine[Title/Abstract] OR dihydrocodeine[Title/Abstract] OR norgesic[Title/Abstract] OR temgesic[Title/Abstract] | #2 |
| **365,642** | alcohol drinking[MeSH Terms] OR alcohol*[Title/Abstract] | #3 |
| **394,837** | street drugs[MeSH Terms] OR street drug*[Title/Abstract] OR recreational drug*[Title/Abstract] OR illicit*[Title/Abstract] OR substance misuse[Title/Abstract] OR drug misuse[Title/Abstract] OR substance us*[Title/Abstract] OR drug us*[Title/Abstract] OR substance abuse*[Title/Abstract] OR substance dependen*[Title/Abstract] OR drug abuse*[Title/Abstract] OR drug dependen*[Title/Abstract] OR substance use disorder*[Title/Abstract] OR addict*[Title/Abstract] OR substance-related disorders[MeSH Terms] | #4 |
| **852,021** | #1 OR #2 OR #3 OR #4 | #5 |
| **66,557** | Iran[MeSH Terms] OR Iran*[Title/Abstract] OR Persia*[Title/Abstract] OR Tehran[Title/Abstract] OR Teheran[Title/Abstract] OR Babol[Title/Abstract] OR Tabriz[Title/Abstract] OR Rasht[Title/Abstract] OR Mashhad[Title/Abstract] OR Mashad[Title/Abstract] OR Zahedan[Title/Abstract] OR Shiraz[Title/Abstract] OR Ahvaz[Title/Abstract] OR Isfahan[Title/Abstract] OR Esfahan[Title/Abstract] OR Yasouj[Title/Abstract] OR Yasuj[Title/Abstract] OR Arak[Title/Abstract] OR Kerman[Title/Abstract] OR Kermanshah[Title/Abstract] OR Rafsanjan[Title/Abstract] OR Bakhtaran[Title/Abstract] OR Urmia[Title/Abstract] OR Orumieh[Title/Abstract] OR Oroomieh[Title/Abstract] OR Mazandaran[Title/Abstract] OR Gilan[Title/Abstract] OR Guilan[Title/Abstract] OR Guillan[Title/Abstract] OR Gillan[Title/Abstract] OR Semnan[Title/Abstract] OR Yazd[Title/Abstract] OR Hormozgan[Title/Abstract] OR Kohgiluye[Title/Abstract] OR Kohkiluye[Title/Abstract] OR Sanandaj[Title/Abstract] OR Qazvin[Title/Abstract] OR Sabzevar[Title/Abstract] OR Ardabil[Title/Abstract] OR Ardebil[Title/Abstract] OR Bushehr[Title/Abstract] OR Booshehr[Title/Abstract] OR Boushehr[Title/Abstract] OR Ilam[Title/Abstract] OR Golestan[Title/Abstract] OR Gorgan[Title/Abstract] OR Kordestan[Title/Abstract] OR Kurdistan[Title/Abstract] OR Kordistan[Title/Abstract] OR Kurdestan[Title/Abstract] OR Karaj[Title/Abstract] OR Shahrekord[Title/Abstract] OR Jahrom[Title/Abstract] OR Shahroud[Title/Abstract] OR Shahrud*[Title/Abstract] OR Shahrood[Title/Abstract] OR Kashan[Title/Abstract] OR Hamedan[Title/Abstract] OR Hamadan[Title/Abstract] OR Zanjan[Title/Abstract] OR Birjand[Title/Abstract] OR Tonekabon[Title/Abstract] OR Lorestan[Title/Abstract] OR Khoramabad[Title/Abstract] OR Khorramabad[Title/Abstract] OR Alborz[Title/Abstract] OR Khorasan[Title/Abstract] | #6 |
| **3,932,477** | epidemiologic studies [MeSH Terms] OR epidemiology [MeSH Terms] OR epidemiolog* [Title/Abstract] OR prevalence [MeSH Terms] OR prevalence [Title/Abstract] OR incidence [MeSH Terms] OR incidence [Title/Abstract] | #7 |
| **1,261** | #5 AND #6 AND #7 | #8 |
| **799** | #8 AND Filter: Humans | #9 |
| **779** | #8 AND Filters: Publication date from 1990/01/01; Humans | #10 |

**The search strategy used in the Scopus database - Searched at 16 March 2021**

| **Items found** | **Search query** |  |
| --- | --- | --- |
| **290,530** | TITLE-ABS-KEY ( cannabis ) OR TITLE-ABS-KEY ( cannabi* ) OR TITLE-ABS-KEY ( marijuana ) OR TITLE-ABS-KEY ( stimulant ) OR TITLE-ABS-KEY ( stimulant* ) OR TITLE-ABS-KEY ( amphetamine ) OR TITLE-ABS-KEY ( amphetamin* ) OR TITLE-ABS-KEY ( methamphetamine ) OR TITLE-ABS-KEY ( methamphetamin* ) OR TITLE-ABS-KEY ( "Methyl amphetamine" ) OR TITLE-ABS-KEY ( "methyl amphetamin*" ) OR TITLE-ABS-KEY ( ephedrine ) OR TITLE-ABS-KEY ( ephedrin* ) OR TITLE-ABS-KEY ( deoxyephedrine ) OR TITLE-ABS-KEY ( "deoxy ephedrine" ) OR TITLE-ABS-KEY ( lsd ) OR TITLE-ABS-KEY ( "lysergic acid" ) OR TITLE-ABS-KEY ( cocaine ) OR TITLE-ABS-KEY ( cocain* ) OR TITLE-ABS-KEY ( ecstasy ) OR TITLE-ABS-KEY ( phencyclidine ) OR TITLE-ABS-KEY ( phencyclidin* ) OR TITLE-ABS-KEY ( pcp ) OR TITLE-ABS-KEY ( hallucinogen ) OR TITLE-ABS-KEY ( hallucinogen* ) | #1 |
| **369,241** | ( TITLE-ABS-KEY ( opium )  OR  TITLE-ABS-KEY ( morphine )  OR  TITLE-ABS-KEY ( morphin* )  OR  TITLE-ABS-KEY ( codeine )  OR  TITLE-ABS-KEY ( codein* )  OR  TITLE-ABS-KEY ( methadone )  OR  TITLE-ABS-KEY ( methadon* )  OR  TITLE-ABS-KEY ( narcotic* )  OR  TITLE-ABS-KEY ( narcotic )  OR  TITLE-ABS-KEY ( heroin )  OR  TITLE-ABS-KEY ( opioid )  OR  TITLE-ABS-KEY ( opioid* )  OR  TITLE-ABS-KEY ( opiat* )  OR  TITLE-ABS-KEY ( opiate )  OR  TITLE-ABS-KEY ( tramadol )  OR  TITLE-ABS-KEY ( tramadol* )  OR  TITLE-ABS-KEY ( hydromorphon )  OR  TITLE-ABS-KEY ( diphenoxylate )  OR  TITLE-ABS-KEY ( pentazocine )  OR  TITLE-ABS-KEY ( buprenorphine )  OR  TITLE-ABS-KEY ( buprenorphin* )  OR  TITLE-ABS-KEY ( dihydrocodeine )  OR  TITLE-ABS-KEY ( norgesic )  OR  TITLE-ABS-KEY ( temgesic ) ) | #2 |
| **1,617,056** | TITLE-ABS-KEY ( "substance related disorders" ) OR TITLE-ABS-KEY ( street AND drug* ) OR TITLE-ABS-KEY ( "recreational drug*" ) OR TITLE-ABS-KEY ( illicit ) OR TITLE-ABS-KEY ( illicit* ) OR TITLE-ABS-KEY ( "substance abuse" ) OR TITLE-ABS-KEY ( substance AND abus* ) OR TITLE-ABS-KEY ( substance AND use* ) OR TITLE-ABS-KEY ( "drug use*" ) OR TITLE-ABS-KEY ( substance AND misuse ) OR TITLE-ABS-KEY ( drug AND misuse ) OR TITLE-ABS-KEY ( substance AND dependen* ) OR TITLE-ABS-KEY ( "substance dependence" ) OR TITLE-ABS-KEY ( "drug abuse" ) OR TITLE-ABS-KEY ( drug AND abus* ) OR TITLE-ABS-KEY ( drug AND dependen* ) OR TITLE-ABS-KEY ( "drug dependence" ) OR TITLE-ABS-KEY ( "substance use disorders" ) OR TITLE-ABS-KEY ( substance AND use AND disorder* ) OR TITLE-ABS-KEY ( addict* ) OR TITLE-ABS-KEY ( addiction ) | #3 |
| **919,963** | TITLE-ABS-KEY ( alcohol* ) | #4 |
| **231,100** | TITLE-ABS-KEY ( iran* ) OR TITLE-ABS-KEY ( persia* ) OR TITLE-ABS-KEY ( tehran ) OR TITLE-ABS-KEY ( teheran ) OR TITLE-ABS-KEY ( babol ) OR TITLE-ABS-KEY ( tabriz ) OR TITLE-ABS-KEY ( rasht ) OR TITLE-ABS-KEY ( mashhad ) OR TITLE-ABS-KEY ( mashad ) OR TITLE-ABS-KEY ( zahedan ) OR TITLE-ABS-KEY ( shiraz ) OR TITLE-ABS-KEY ( ahvaz ) OR TITLE-ABS-KEY ( isfahan ) OR TITLE-ABS-KEY ( esfahan ) OR TITLE-ABS-KEY ( yasouj ) OR TITLE-ABS-KEY ( yasuj ) OR TITLE-ABS-KEY ( arak ) OR TITLE-ABS-KEY ( kerman ) OR TITLE-ABS-KEY ( kermanshah ) OR TITLE-ABS-KEY ( rafsanjan ) OR TITLE-ABS-KEY ( bakhtaran ) OR TITLE-ABS-KEY ( urmia ) OR TITLE-ABS-KEY ( orumieh ) OR TITLE-ABS-KEY ( oroomieh ) OR TITLE-ABS-KEY ( mazandaran ) OR TITLE-ABS-KEY ( gilan ) OR TITLE-ABS-KEY ( guilan ) OR TITLE-ABS-KEY ( guillan ) OR TITLE-ABS-KEY ( gillan ) OR TITLE-ABS-KEY ( semnan ) OR TITLE-ABS-KEY ( yazd ) OR TITLE-ABS-KEY ( hormozgan ) OR TITLE-ABS-KEY ( kohgiluye ) OR TITLE-ABS-KEY ( kohkiluye ) OR TITLE-ABS-KEY ( sanandaj ) OR TITLE-ABS-KEY ( qazvin ) OR TITLE-ABS-KEY ( sabzevar ) OR TITLE-ABS-KEY ( ardabil ) OR TITLE-ABS-KEY ( ardebil ) OR TITLE-ABS-KEY ( bushehr ) OR TITLE-ABS-KEY ( booshehr ) OR TITLE-ABS-KEY ( boushehr ) OR TITLE-ABS-KEY ( ilam ) OR TITLE-ABS-KEY ( golestan ) OR TITLE-ABS-KEY ( gorgan ) OR TITLE-ABS-KEY ( kordestan ) OR TITLE-ABS-KEY ( kurdistan ) OR TITLE-ABS-KEY ( kordistan ) OR TITLE-ABS-KEY ( kurdestan ) OR TITLE-ABS-KEY ( karaj ) OR TITLE-ABS-KEY ( shahrekord ) OR TITLE-ABS-KEY ( jahrom ) OR TITLE-ABS-KEY ( shahroud ) OR TITLE-ABS-KEY ( shahrud* ) OR TITLE-ABS-KEY ( shahrood ) OR TITLE-ABS-KEY ( kashan ) OR TITLE-ABS-KEY ( hamedan ) OR TITLE-ABS-KEY ( hamadan ) OR TITLE-ABS-KEY ( zanjan ) OR TITLE-ABS-KEY ( birjand ) OR TITLE-ABS-KEY ( tonekabon ) OR TITLE-ABS-KEY ( lorestan ) OR TITLE-ABS-KEY ( khoramabad ) OR TITLE-ABS-KEY ( khorramabad ) OR TITLE-ABS-KEY ( alborz ) OR TITLE-ABS-KEY ( khorasan ) | #5 |
| **2,783,921** | TITLE-ABS-KEY ( epidemiologic AND studies ) OR TITLE-ABS-KEY ( epidemiology ) OR TITLE-ABS-KEY ( epidemiolog* ) OR TITLE-ABS-KEY ( prevalence ) OR TITLE-ABS-KEY ( incidence ) | #6 |
| **2,803,595** | #1 OR #2 OR #3 OR #4 | #7 |
| **2,188** | #5 AND #6 AND #7 | #8 |
| **2,159** | #8 AND PUBYEAR AFT 1990 | #9 |

**The search strategy used in the ISI database - Searched at 16 March 2021**

| **Items found** | **Search query** | **#** |
| --- | --- | --- |
| **183,538** | TS=( cannabi* OR cannabis OR marijuana OR stimulant OR stimulant* OR amphetamine OR amphetamin* OR methamphetamine OR methamphetamin*OR Methylamphetamine OR methylamphetamin* OR ephedrine OR ephedrin* OR deoxyephedrine OR mdeoxyephedrin* OR LSD OR (lysergic acid) OR cocaine OR cocain* OR ecstasy OR phencyclidin* OR phencyclidine OR PCP OR Hallucinogen OR Hallucinogen*) Indexes=SCI-EXPANDED, SSCI, A&HCI, CPCI-S, CPCI-SSH, BKCI-S, BKCI-SSH, ESCI, CCR-EXPANDED, IC Timespan=1990-2021 | #1 |
| **180,082** | TS=(opium OR morphine OR morphin* OR codeine OR codein* OR methadone OR methadon* OR narcotic* OR narcotic OR heroin OR opioid OR opioid* OR opiat* OR opiate OR tramadol OR tramadol* OR hydromorphone OR meperidine OR dihydromorphine OR diphenoxylate OR Pentazocine OR buprenorphine OR buprenorphin* OR dihydrocodeine OR Norgesic OR temgesic) Indexes=SCI-EXPANDED, SSCI, A&HCI, CPCI-S, CPCI-SSH, BKCI-S, BKCI-SSH, ESCI, CCR-EXPANDED, IC Timespan=1990-2021 | #2 |
| **542,837** | TS=(alcohol*) Indexes=SCI-EXPANDED, SSCI, A&HCI, CPCI-S, CPCI-SSH, BKCI-S, BKCI-SSH, ESCI, CCR-EXPANDED, IC Timespan=1990-2021 | #3 |
| **1,096,982** | TS=((substance related disorder*) OR (street drug*) OR (recreational drug*) OR illicit* OR (substance abus*) OR (substance misuse) OR (drug use*) OR (drug misuse) OR (substance use*) OR (substance dependen*) OR (drug abus*) OR (drug dependen*) OR (substance use disorder*) OR addict*) Indexes=SCI-EXPANDED, SSCI, A&HCI, CPCI-S, CPCI-SSH, BKCI-S, BKCI-SSH, ESCI, CCR-EXPANDED, IC Timespan=1990-2021 | #4 |
| **1,768,675** | #4 OR #3 OR #2 OR #1 Indexes=SCI-EXPANDED, SSCI, A&HCI, CPCI-S, CPCI-SSH, BKCI-S, BKCI-SSH, ESCI, CCR-EXPANDED, IC Timespan=1990-2021 | #5 |
| **135,847** | TS=(Iran* OR Persia* OR Tehran OR Teheran OR Babol OR Tabriz OR Rasht OR Mashhad OR Mashad OR Zahedan OR Fars OR Shiraz OR Ahvaz OR Isfahan OR Esfahan OR Yasouj OR Yasuj OR Arak OR Kerman OR Kermanshah OR Rafsanjan OR Bakhtaran OR Urmia OR Orumieh OR Oroomieh OR Mazandaran OR Gilan OR Guilan OR Guillan OR Gillan OR Semnan OR Yazd OR Hormozgan OR Kohgiluye OR Kohkiluye OR Sanandaj OR Qazvin OR Sabzevar OR Ardabil OR Ardebil OR Bushehr OR Booshehr OR Boushehr OR Ilam OR Golestan OR Gorgan OR Kordestan OR Kurdistan OR Kordistan OR Kurdestan OR Karaj OR Shahrekord OR Jahrom OR Shahroud OR Shahrud* OR Shahrood OR Kashan OR Hamedan OR Hamadan OR Zanjan OR Birjand OR Tonekabon OR Lorestan OR Khoramabad OR Khorramabad OR Alborz OR Khorasan) Indexes=SCI-EXPANDED, SSCI, A&HCI, CPCI-S, CPCI-SSH, BKCI-S, BKCI-SSH, ESCI, CCR-EXPANDED, IC Timespan=1990-2021 | #6 |
| **1,913,475** | TS=((epidemiologic studies) OR epidemiology OR epidemiolog* OR prevalence OR incidence) Indexes=SCI-EXPANDED, SSCI, A&HCI, CPCI-S, CPCI-SSH, BKCI-S, BKCI-SSH, ESCI, CCR-EXPANDED, IC Timespan=1990-2021 | #7 |
| **1,852** | #7 AND #6 AND #5 Indexes=SCI-EXPANDED, SSCI, A&HCI, CPCI-S, CPCI-SSH, BKCI-S, BKCI-SSH, ESCI, CCR-EXPANDED, IC Timespan=1990-2021 | #8 |
