# Supplementary material for: Evidence for an increase in cannabis use in Iran – A systematic review and trend analysis
Source: PLoS One. 2021 Aug 30;16(8):e0256563. doi: 10.1371/journal.pone.0256563 (PMC8404985; doi:10.1371/journal.pone.0256563)
Supplement: S2 Table — (DOCX) [file pone.0256563.s013.docx]

### S2 Table - Quality assessment tools

|  | **Items** | **Low risk of bias** | **High risk of bias** |
| --- | --- | --- | --- |
| 1 | Was the source of sampling well presented? Was the sample representative of the target population? | Representative | Otherwise or unclear. |
| 2 | Was the method of sampling appropriate? | Random or census; Multistage method for studies among the high-risk population. | Otherwise or unclear. |
| 3 | Was the sample size adequate? | Adequate (more than 30). | Otherwise or unclear. |
| 4 | Were the study subjects and the setting described in detail? | Well described. | Not described or unclear. |
| 5 | Was the year of the study stated? | Reported. | Not reported. |
| 6 | Was the response rate provided and was it over 70%? If below 70%, were the non-responders not different from respondents in main demographic characteristics? | Response rate upper than 70% or under 70% but the non-responders not different from respondents in main demographic characteristics | Otherwise or unclear. |
| 7 | Was the condition measured by valid method? | Reliable and valid questionnaire. | Otherwise or unclear. |
| 8 | Were standard criteria used for the measurement of the condition? | The definition of use and the indicator of the time were presented. | Not reported or unclear. |
| 9 | Were subgroup analyses for sex, recruitment setting, the definition of use, or time indicator performed? | Performed. | Not performed |
