# Supplementary material for: Evidence for an increase in cannabis use in Iran – A systematic review and trend analysis
Source: PLoS One. 2021 Aug 30;16(8):e0256563. doi: 10.1371/journal.pone.0256563 (PMC8404985; doi:10.1371/journal.pone.0256563)
Supplement: S3 Table — (DOCX) [file pone.0256563.s014.docx]

### S3 Table – Meta-regression of possible sources of heterogeneity

| **Study level covariates** | **Coefficient** | **95% CI** | | **P value** |
| --- | --- | --- | --- | --- |
| Sex (male vs. female) | 0.11 | 0.08 | 0.13 | *<0.001* |
| Indicator |  |  |  |  |
| Last 12-month vs. lifetime | -0.07 | -0.13 | -0.14 | *0.01* |
| Last month or current vs. lifetime | -0.05 | -0.10 | -0.04 | 0.07 |
| Daily or almost daily vs. lifetime | -0.12 | -0.19 | -0.06 | *<0.001* |
| Study year | -0.001 | -0.005 | 0.003 | 0.69 |
| Number of unfulfilled quality criteria | 0.06 | 0.03 | 0.08 | *<0.0001* |
| Participants |  |  |  |  |
| High school vs. general population | 0.04 | -0.02 | 0.10 | 0.17 |
| University vs. general population | 0.03 | -0.01 | 0.09 | 0.08 |
| Young general population vs. general population | 0.04 | -0.01 | 0.10 | 0.11 |
| High-risk population vs. general population | 0.33 | 0.26 | 0.40 | *<0.0001* |
| Age group (combined youth groups, including young general population, university and high school students, vs. general population) | 0.04 | 0.01 | 0.08 | *0.01* |
