# Supplementary material for: Evidence for an increase in cannabis use in Iran – A systematic review and trend analysis
Source: PLoS One. 2021 Aug 30;16(8):e0256563. doi: 10.1371/journal.pone.0256563 (PMC8404985; doi:10.1371/journal.pone.0256563)
Supplement: S4 Table — (DOCX) [file pone.0256563.s015.docx]

### S4 Table – Trends of various cannabis use measures among the "combined youth groups" and national cannabis seizures

| **Indicator** | **Coefficient for linear trend**  **(95% CI)** | **P value** |
| --- | --- | --- |
| **Combined youth groups - Male** |  |  |
| Lifetime | 0.004(-0.001 – 0.008) | 0.127 |
| Last 12 months | 0.05(0.0003 – 0.010) | *0.035* |
| Last month or current | 0.004(-0.002 – 0.009) | 0.181 |
| Daily or almost daily | -0.004(-0.008 – 0.0003) | 0.072 |
| **Combined youth groups - Female** |  |  |
| Lifetime | 0.0002(-0.004 – 0.004) | 0.929 |
| Last 12 months | 0.002(-0.004 – 0.007) | 0.565 |
| Last month or current | 0.002(-0.001 – 0.005) | 0.176 |
| Daily or almost daily | -0.002(-0.004 – 0.001) | 0.083 |
| **National seizures of cannabis** |  |  |
| Last year | 0.402(0.338-0.467) | *<0.001* |
