# Supplementary material for: Evidence for an increase in cannabis use in Iran – A systematic review and trend analysis
Source: PLoS One. 2021 Aug 30;16(8):e0256563. doi: 10.1371/journal.pone.0256563 (PMC8404985; doi:10.1371/journal.pone.0256563)
Supplement: Attachment — Submitted filename: Response to Reviewers.docx [file pone.0256563.s018.docx]

**Reviewers' comments**

**Reviewer #1:**

**All comments have been addressed.**

**Reviewer #2:**

**Dear authors,**

**Thank you for addressing my previous comments and for providing clarifications. I appreciate your effort in synthesizing data and providing a comprehensive and up-to-date review of cannabis use in Iran. I provided additional suggestions that could increase the value of the manuscript. I encourage the authors to properly acknowledge the contribution of the work of Nazarzadeh et al., considering the paucity of reviews related to cannabis use in Iran and to pay more attention to how the methodology is reported e.g., lack of research questions, incomplete eligibility and exclusion criteria. I am well aware of the challenges associated with conducting and publishing systematic reviews and I hope that the authors will consider the suggestions provided below.**

Many thanks for the comprehensive review and insightful comments. Certainly, they would significantly improve this study. We have applied and responded to all the comments:

**1) In my original report, I suggested that the authors remove from the abstract/results the analyses related to national cannabis seizures as this is not an objective of the review and keep this topic for the discussion section. The authors responded, “We omitted seizures' related sentence from the Result section of the abstract” but I am unsure why they kept following statement in the abstract “Trends of various use indicators and national seizures were examined.”**

We omitted this statement from the Abstract.

**2) In the abstract, following statement comes out of the blue: “Treatment seeking for cannabis use disorder among those w­ith substance use disorder attending treatment ranged from 0.9% to 10.9%”.**

We omitted this sentence from the Abstract.

**3) In the introduction, the authors inaccurately state “Several studies have examined the prevalence of cannabis use along with other drugs in the general population; however, we know little about the prevalence of cannabis use in different Iranian population subgroups.” In my opinion, the authors should adequately acknowledge in the introduction and discussion sections the systematic review published by Nazarzadeh et al., (2015). Prevalence of Cannabis Lifetime Use in Iranian High School and College Students: A Systematic Review, Meta-Analyses, and Meta-Regression. DOI: 10.1177/1557988314546667. In this review, the authors provide more data related to cannabis use/dependence (by including more groups such as general population, high-risk populations) compared to the review conducted by Nazardeh et al. Consequently, I encourage the authors to elaborate on similarities and differences such as the number of studies retained (and number of participants) in the university and high-school groups, lifetime cannabis use, etc.**

The relevant section in the Introduction was edited accordingly, pointing to the differences in the scope of the two studies. Moreover, the result of the previous study was added in more detail in the Discussion section.

**4) In my opinion, the authors should provide a clear description of the eligibility criteria. I recommend that the authors provide in a dedicated paragraph (and not in the data extraction section) a clear description of the population of interest (e.g., what “general population means”) and of the outcomes. The authors provided in the PRISMA flow diagram exclusion criteria such as “non-eligible source population” but this source population was not defined in the eligibility criteria section.**

We moved the mentioned sentences to the "Eligibility criteria and screening" section as a separate paragraph and extended the details on the definition of the target population. The "non-eligible source population" was added to the eligibility criteria.

**5) It is important that the authors clearly define what was included in the Cannabis Use Disorder (CUD) outcome. The CUD diagnostic criteria were introduced in DSM-5 (2013) and between 2000-2013 the DSM-4 used the terminology “cannabis dependence” and “Cannabis abuse”. Therefore, using CUD for studies conducted before 2013 is misleading and it would be adequate to define this outcome as cannabis dependence/abuse. In some sections (introduction, discussion) the authors refer to cannabis use disorder, in other sections (e.g., high risk groups) to cannabis dependence.**

The relevant sentence in the Method section was clarified as below:

"Whatever criteria of the cannabis use disorder, either based on Diagnostic and Statistical Manual of Mental Disorders version IV or V or any other definitions, the studies were included. The applied criteria were reported precisely as stated in the study."

We stated whatever terminology the studies applied in the result section and relative tables; however, in the Introduction and Discussion sections we used "cannabis use disorder", to be consistent with the latest DSM-V.

**6) The authors provided in their response the rationale for reporting results based on different population groups i.e., differences between students and other youth. I encourage that the authors explain in the manuscript the rationale for selecting these groups. I am unsure whether the decision to analyze these groups was made before starting the systematic review (as suggested by the study aims provided in the introduction) or at the analysis phase.**

Based on the previous reviews conducted by our center, studies investigating the prevalence of substance use are mainly conducted among the general population, university students, high school students, or high-risk subgroups in Iran. Therefore, we selected the target population accordingly. This rationale was added to the Method section.

**7) The authors excluded from analyses studies that did not report cannabis use separately for men and women, but this is not stated as an exclusion criterion in the PRISMA flow diagram. Therefore, it is difficult for the reader to figure out how many studies were excluded based on this criterion.**

As these studies were not excluded from the systematic review and were presented in the tables, we did not add this criterion to the eligibility criteria. However, we added the number of studies excluded from the meta-analysis to the Result section.

**8) To my understanding the population groups used are mutually exclusive e.g., general population, university students, etc. The sum of studies reported in tables 1 to 4 is 102 and does not correspond to the number of studies included in the review based on the PRISMA diagram (90 studies).**

This is since there are "Four studies provided measures for both the general population and young general population." Also, we have presented the results of four different provinces and rounds of the Persian Youth Cohort study (six rows for one study) and the result of one repeated cross-sectional study among university students (four rows for one study) separately in the relative tables. This would add up to 102 rows for 90 studies. We added two clarifying sentences regarding the latter issue in the Result section.

**9) The authors acknowledged in their response that they did not keep track of the screening process for the 50 studies identified outside of the international databases search. I appreciate their efforts to identify as many eligible studies as possible, but this process lacks transparency and impedes on the reproducibility of the review. Therefore, I consider that this should be acknowledged as a limitation especially because more than half of the total number of included studies were found using this method.**

Was added in the Limitation section.

**10) As previously suggested, it would be useful for readers that the authors provide the name of the R package used for analyzing data. The authors (based on my previous suggestion) provided the name of the functions but omitted the name of the package.**

The name of the packages was added.

**11) As suggested in my report, it would be useful to provide a short description of the “network scale-up (NSU) method” since the authors excluded NSU studies from the analyses and to provide the number of studies that were excluded based on this criterion.**

A brief definition was added in the Method section. The number of studies excluded accordingly was added in the Result section.
